# Fast Radio Map Construction by using Adaptive Path Loss Model Interpolation in Large-Scale Building

**DOI:** 10.3390/s19030712

**Published:** 2019-02-10

**Authors:** Jingxue Bi, Yunjia Wang, Zengke Li, Shenglei Xu, Jiapeng Zhou, Meng Sun, Minghao Si

**Affiliations:** 1 NASG Key Laboratory of Land Environment and Disaster Monitoring, China University of Mining and Technology, Xuzhou 221116, China; bjx1050@163.com; 2 School of Environmental Science and Spatial Informatics, China University of Mining and Technology, Xuzhou 221116, China; zengkeli@yeah.net (Z.L.); tb17160013b2@cumt.edu.cn (S.X.); ts16160038a3@cumt.edu.cn (J.Z.); tb18160007b1@cumt.edu.cn (M.S.); 13218021689@163.com (M.S.)

**Keywords:** radio map, fingerprinting, indoor positioning, least squares, path loss model

## Abstract

The radio map construction is usually time-consuming and labor-sensitive in indoor fingerprinting localization. We propose a fast construction method by using an adaptive path loss model interpolation. Received signal strength (RSS) fingerprints are collected at sparse reference points by using multiple smartphones based on crowdsourcing. Then, the path loss model of an access point (AP) can be built with several reference points by the least squares method in a small area. Afterwards, the RSS value can be calculated based on the constructed model and corresponding AP’s location. In the small area, all models of detectable APs can be built. The corresponding RSS values can be estimated at each interpolated point for forming the interpolated fingerprints considering RSS loss, RSS noise and RSS threshold. Through combining all interpolated and sparse reference fingerprints, the radio map of the whole area can be obtained. Experiments are conducted in corridors with a length of 211 m. To evaluate the performance of RSS estimation and positioning accuracy, inverse distance weighted and Kriging interpolation methods are introduced for comparing with the proposed method. Experimental results show that our proposed method can achieve the same positioning accuracy as complete manual radio map even with the interval of 9.6 m, reducing 85% efforts and time of construction.

## 1. Introduction

The indoor positioning technology attracts extensive attentions of researchers to carry out immense amounts of studies and develop corresponding localization systems, such as Wi-Fi, Bluetooth, pedestrian dead reckoning (PDR/DR), radio frequency identity (RFID), infrared, ultrasonic, Zigbee, magnetic field, visible light, computer vision, and pseudolites [1]. Among them, the fingerprinting method based on received signal strength (RSS) may be the most widely used technique, which is suitable for both Wi-Fi and Bluetooth with low-cost and highly accessible devices. In fact, it is also applied with cellular, RFID or ZigBee signals [2].

The RSS-based fingerprint positioning method includes two steps: A training step and tracking step. During the training step, the main work is to collect RSS fingerprints for constructing a radio map (RM) and yield the mapping relationship between signal fingerprints and spatial positions. The basic idea of the tracking step is to estimate pending location by matching RSS collection with the aforementioned radio map. Radio map generally determines fingerprinting localization accuracy. Therefore, it plays a crucial role in indoor fingerprinting localization. Radio map comprises a large number of location-labeled RSS fingerprints gathered from hearable wireless access points (APs) or iBeacons at specified reference points (RPs). In general, RPs are evenly distributed in the whole interested area. Because radio map is made up of thousands of location-labeled fingerprints, it is also called as fingerprint database. To describe the characteristic of wireless signals accurately, several tens of samples are performed at each RP. Occasionally, to avoid the sheltering impact of the human body on RSS, the procedure requires collectors to face four different directions [3,4]. The efforts and time for collection will greatly increase as the system coverage expands. Obviously, the radio map construction is time-consuming and labor-sensitive, hindering the wide application and promotion of RSS fingerprinting localization. Furthermore, the complex and dynamic wireless indoor environment makes radio map maintenance difficult. Wireless signal, in particular, easily influenced by structures, layout and pedestrians in the building. Although there are many challenges for radio map construction, lots of methods are proposed to solve these issues, such as methods based on crowdsourcing [5,6,7], simultaneous localization and mapping (SLAM) [8,9], inertial sensors [10,11,12], semi-supervised learning [13,14] or unsupervised learning [15,16], path loss model [17,18], and interpolation [19,20,21]. It is worth noting that most of SLAM, inertial sensors, semi-supervised learning methods also belong to crowdsourcing methods.

Crowdsourcing methods can collect RSS samples with a short time period and little workload covering a building, but the collected RSS samples could not characterize their distributions well. The positioning performance of the built radio map by crowdsourcing is usually poor. The radio maps built by interpolation methods usually get good positioning performance, but when the interval of sparse RPs enlarges, the positioning accuracy will decrease. By means of crowdsourcing, it is easy to get several sparse RPs with RSS fingerprints and obtain accurate path loss models in a small area. Therefore, we propose a method of radio map construction by merging crowdsourcing, interpolation and path loss model. This method could get the same positioning accuracy when the interval of sparse RPs is set as 9.6 m as the complete manual radio map with the interval of 1.2 m. The main contributions of this work are summarized as shown below.

- We propose a method of radio map construction by using crowdsourcing, path loss model and interpolation methods, which can greatly reduce the workload and time of radio map construction and ensure the same positioning accuracy as the complete manual radio map. It is also the novelty of the paper.
- Least squares algorithm is utilized for estimating optimal parameters of path loss model. It allows the method adaptively construct path loss models of all detectable APs in a small area. The random noise, RSS loss and threshold restraint are taken into account in RSS calculation based on the path loss model.
- By comparing with inverse distance weighted (IDW) and Kriging interpolation methods, the generated radio map based on our proposed method behaves well in positioning performance. The radio map constructed by only 15% RPs with the interval of 9.6 m can achieve the same positioning accuracy as the complete manual radio map.

The remainder of this paper is organized as follows. In Section 2, several methods for reducing the workload of radio map construction are summarized. Section 3 describes the motivation to study the method of radio map construction. Section 4 presents the method of radio map construction by using crowdsourcing and path loss model interpolation in detail. Section 5 introduces the experimental testbed, a series of comparison experiments with IDW, Kriging, our proposed interpolation radio maps and the complete manual one. A discussion is conducted in Section 6. Conclusions are drawn in Section 7.

## 2. Related Work

In this section, several existing methods for radio map construction are introduced to compare with each other. A lot of methods are proposed to reduce radio map building efforts for RSS fingerprint-based localization, such as methods based on crowdsourcing, SLAM, inertial sensors, semi-supervised or unsupervised learning, interpolation and path loss model. Because SLAM-based, inertial sensors-based, semi-supervised learning, unsupervised learning and interpolation methods are usually used in cooperation with the crowdsourcing method, there is no clear distinction between them. Comparisons are summarized in Table 1, where HMM, EM, ME, MD, STD represent hidden markov model, expectation maximization, mean, median, and standard deviation, respectively.

The basic idea of crowdsourcing is assigning workload to multiple participants, who are not only professional surveyors but also common users. Crowdsourcing can be divided into two categories based on the user’s intervention: Active and passive crowdsourcing [22]. Redpin [5], Molé [6] and FreeLoc [7] all belong to active crowdsourcing methods. They have been developed for prompting users to provide fingerprint measurements with locations or semantic labels, such as rooms, hallways, and corridors. But users are usually reluctant to provide accurate location labels, which seriously affect the performance of the built radio map. The passive or implicit crowdsourcing methods are proposed based on trajectories or reference points. Most of the SLAM-based and inertial sensors-based methods belong to passive crowdsourcing methods. Meanwhile, semi-supervised learning method can greatly reduce the workload in cooperation with crowdsourcing method. However, the method makes the device heterogeneity very serious because of millions of different smart phones. Training and tracking devices are likely to be different, affecting the performance of fingerprinting localization. Therefore, location labels and device heterogeneity are the big challenges for crowdsourcing methods.

Methods that are based on SLAM and inertial sensors leverage human motions or trajectories to provide location labels and simultaneously collect RSS fingerprints. WiFi-SLAM [8] uses the Gaussian process latent variable models (GP-LVM) to relate RSS fingerprints with human movements. SignalSLAM [9] can simultaneously collect time-stamped Wi-Fi and Bluetooth RSS, 4G LTE signal strength information and magnetic field magnitude, providing real-time location by using a modified version of GraphSLAM optimization. Both WiFi-SLAM and SignalSLAM are not able to operate on smartphones. SmartSLAM [23] is the first SLAM-based system running on smartphones, estimating locations based on the hidden Markov model and particle filter, constructing an indoor map by extended Kalman filter. At the same time, it gives some discussions about radio map construction. Zee [10], LiFS [11] and WILL [12] provide location labels by using the PDR algorithm. In the PDR algorithm, the accelerometer is used for step counting; magnetic field and gyroscope are fused to estimate orientation angle. PDR makes real-time providing locations possible based on the embedded inertial sensors in smartphones. The indoor map is also introduced for obtaining precise locations by map constraint or features extraction [10,11], such as landmarks and obstacles. Nevertheless, positions provided by SLAM and PDR are usually with large errors, because of accumulation error of noisy sensors and features mismatching. Although SLAM-based and inertial sensors-based methods can reduce the efforts and time of constructing radio map to some extent, there are still new challenges to overcome, such as sensors drift, orientation estimation, features extraction, device heterogeneity and power consumption.

Semi-supervised and unsupervised learning methods are also employed in radio map construction. Semi-supervised learning method transforms fingerprints to geographic coordinates based on the mapping of unlabeled and partial location-labeled RSS fingerprints [13,14]. Manifold learning [13] and Co-Forest [14] are the used semi-supervised methods, respectively, which construct a non-linear projection that maps high-dimensional signal fingerprints onto a two-dimensional space. As obtaining a large number of unlabeled fingerprints through passive crowdsourcing is quite easy, so the semi-supervised method appears to be a promising solution. However, these semi-supervised methods still require lots of location-labeled fingerprints for initializing the learning process, which takes a long period of time. Jung [15,16] proposes an unsupervised learning method based on a hybrid global-local optimization scheme. This method estimates the optimal position of fingerprints on an indoor map, under the constraint by the inner structure of the map, such as walls and partitions. The indoor space is divided as a set of finite location-states and constrained transitions where each state corresponds to a physical location. If the building is symmetrical, there are multiple mappings between the model and space, failing in model learning. Besides, a radio propagation model is used for addressing optimization problem, but such a propagation model may be not precise enough to describe a dynamic and sophisticated indoor space.

Path loss model generation is another solution to construct a radio map. ARIADNE [17] constructs a new path loss model, which integrates the ray tracing technique, ignores diffraction and scattering effect. It estimates the optimal value of parameters by simulated annealing algorithm and several RPs. Then, ARIADNE can generate radio map by the constructed path loss model with the knowledge of APs’ locations and indoor map. Multi-wall model (MWM) [18,19] is adopted to build a radio map taking the wall attention factor into account. For path loss model methods, if the structure and layout are not changed in the indoor space, each generated radio map will be exactly identical. Obviously, it is not realistic because of the indoor dynamic wireless environment. In addition, it is a big challenge to build a refined path loss model in a certain indoor environment. It seems that ARIADNE can build accurate path loss model based on several RPs, however, ray tracing is only operating on special devices instead of universal smartphones. Moreover, such methods based on the path loss model are explored in a small area with a few APs.

An interpolation is a mathematical tool that can estimate the value at a specified point based on the spatial relationship between nearby points. The IDW and Kriging methods are most widely used for building radio map with approximate positioning accuracy. Literatures [19,24] show that IDW interpolation method can get an acceptable positioning accuracy with a large scale of testbed area and many APs. Kriging [20] method utilizes RPs to formulate a function for yielding unbiased RSS estimation with the minimum error variance at each interpolated point (IP). The RSS value is the best linear unbiased estimation based on the semivariogram, which is calculated by RSS values and positions of several RPs. The cubic spline interpolation method is also used for estimating RSS. Peng [21] adopts the cubic spline interpolation method based on boundary optimization to compute space-related RSS. The testing mean localization accuracy is about 2.95 m with 50% percentile of RPs, while the positioning accuracy of the complete manual radio map is 2.77 m. Zhou [25] uses cubic spline interpolation method to enrich the radio map with the limited number of RPs. In fact, the density of RPs in their area is very high. The sample interval is about 0.65 m. The positioning accuracy of the radio map generated by cubic spline interpolation method is from 2.27 m to 3.21 m with 41% percentage of RPs. In other words, the interpolated interval is about 1.2 m. It seems that interpolation methods might be the simplest solution to construct the radio map, as long as providing some reference points. However, these interpolation methods are unable to estimate RSS fingerprint accurately, in particular, with a large sample interval and many APs. 

## 3. Motivation

SLAM-based or inertial sensors assisted methods can greatly reduce workload and time of radio map construction, but these methods are with poor fingerprinting localization accuracy listed in Table 1, while the path loss model and interpolation methods are with better positioning performance. For the former methods, it is very difficult to seamlessly cover the entire testbed, because of the users’ random trajectories. It seems like that SLAM-based or inertial sensors assisted methods are better suited for radio map update. Therefore, we combine the path loss model and interpolation methods for fast and accurate radio map construction.

As mentioned above, it is a big challenge to build a refined path loss model in a certain large-scale indoor environment. But an accurate path loss model is within easy reach depending on several RPs in a small area. Many pieces of research have studied the path loss model construction based on least squares and RSS fingerprints generation. They have ignored the RSS fluctuation, RSS loss and threshold restraint in RSS estimation, and these studies are covering a small area with few APs and small interval between RPs.

**Figure 1:**
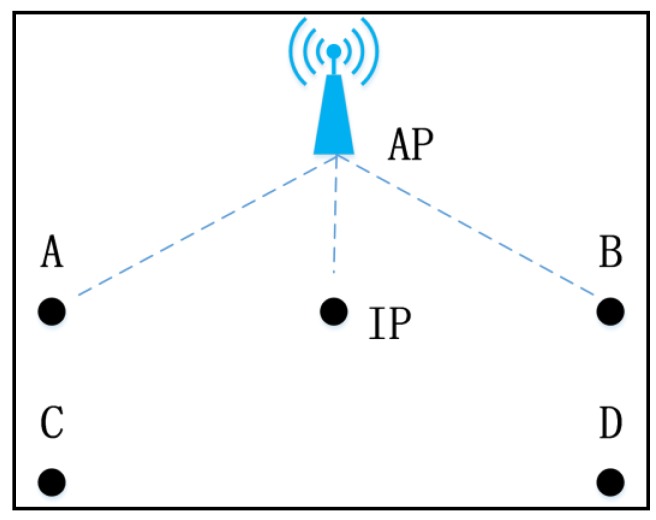
The unsuitable situation for the existing interpolation methods in RSS estimation.

The existing IDW and Kriging interpolation methods usually implement by calculating the weighted mean RSS value of different nearby RPs. They can be described as Equation (1), where *n* is the number of RPs, *R_ref i_* denotes the RSS of *i*th RP, *ω_i_* denotes the weight of *i*th RP, *R*_inter_ denotes the RSS of IP. For IDW method, *ω_i_* are usually set as the inverse of distance squares or distance between *i*th RP and IP. For the Kriging method, *ω_i_* are the best linear unbiased estimation based on semivariance. For these interpolation methods, the RSS value of an AP at IP is a weighted average of RSS values of nearby RPs, and the calculated RSS value is ranging from the minimum and maximum RSS values of RPs. The RSS estimation based on these interpolation methods is not accurate in the situation, shown in Figure 1.

(1) $$R_{\mathrm{inter}}=\sum_{i=1}^{n}\left(\omega_{i}\cdot R_{refi}\right)$$

In Figure 1A–D four points refer to RPs, IP denotes interpolated point, IP is closer to AP than four RPs. In this situation, RSS value received from the AP at IP would be larger than corresponding values at four RPs. No matter which interpolation method is adopted or how weights are allocated, we are unable to get a larger value than the maximum RSS of four RPs by Equation (1). When the interval between RPs becomes larger, the RSS estimation will be more inaccurate, compared with the real RSS value. These interpolation methods seem to be not applicable. Especially, the strong RSS values play more important roles than weak ones in fingerprinting localization.

Therefore, we explore the fast and accurate radio map construction with many APs in a large-scale building, considering RSS fluctuation, RSS loss and RSS threshold. At the same time, we want to find a suitable sample interval between two nearby RPs, which can minimize the construction workload and time with the same positioning accuracy as the complete manual radio map with the interval of 1.2 m.

## 4. Proposed Interpolation Method based on Adaptive Path Loss Model

In this section, RSS fingerprints collected by crowdsourcing will not be described in detail. Because device heterogeneity is a big challenge for the field of indoor positioning and indoor navigation, the effect of device heterogeneity is not taken into account. Before describing the proposed method, we assume that the sparse RPs and APs’ locations are known. In our particular case, approximate locations of APs can be quickly acquired by clicking on the CAD floor plan.

### 4.1. Log-Distance Path Loss Model

A lot of path loss models are summarized in previous work [26,27], such as log-distance, multi-slope, COST231, international telecommunication union (ITU) models. In addition, some modified models are put forward for different indoor scenarios. For example, the multi walls multi floors (MWMF) model with floor and wall attenuation factor is improved based on the log-distance path loss model considering the effects of floors, soft partitions and walls between APs and mobile devices. Alshami [18] adopts the MWMF model to generate dynamic indoor radio map. Liu [28] employs the log-distance path loss model to analyze the effects on positioning accuracy in a small and ideal room. Tao [29] utilizes the log-distance path loss model and Gaussian process regression to infer APs’ locations.

For convenience, we adopt the log-distance path loss model ignoring walls and floors attenuation to construct radio map, as shown in Equation (2). Where *R_d_* is the RSS value at the distance of *d* from the given AP, *R*_*d*0_ is the RSS value at the distance of *d*_0_, *d*_0_ is usually chosen as 1 meter, *n* is the attenuation factor, and *X_σ_* is a noise with zero mean value and standard deviation *σ*.

(2) $$R_{d}=R_{d_{0}}-10n\log_{10}\left(\frac{d}{d_{0}}\right)+X_{\sigma }$$

The simplification of the adopted log-distance path loss model is shown in Equation (3). We use *A* for representing RSS value at distance *d*_0_. As a result, the log-distance path loss model becomes a function of the variable *d*, with two unknown parameters *A* and *n*.

(3) $$R_{d}=A-10n\log_{10}\left(d\right)+X_{\sigma }$$

### 4.2. Parameters Estimation by Least Squares

It is very difficult to build a refined signal propagation model in a whole indoor space, because of unpredictable radio channel attenuation, reflections, diffractions, scatterings, and pedestrians walking. But an accurate path loss model of an AP in a small area can be easily built depending on several RPs. The problem becomes the optimization of parameters estimation transforming from model construction by Equation (3). The least squares are a very simple and effective optimization method based on minimal variance. It can be used for fast, accurate and adaptive path loss model construction.

Equation (3) can be further modified as Equation (4), which can be changed into vector form.

(4) $$X_{\sigma }=A-10n\log_{10}\left(d\right)-R_{d}$$

(5) $$X_{\sigma }=\left[\begin{array}{cc} 1 & -10\log_{10}d \end{array}\right]\left[\begin{array}{c} A\\ n \end{array}\right]-R_{d}$$

We assume that there are *m* adjacent RPs in the small area, *m* is larger than 2. *m* equations can be built as Equation (6), where *R_dm_* denotes RSS noise received at the *m*th RPs from certain AP, *X_σm_* refers to RSS noise, *d_m_* is the distance between the RP and certain AP. Assuming that the RSS measurements are independent, the weight matrix *P* is set as a unit matrix with *m* × *m* dimensions. Then, we can estimate the above-mentioned two parameters by Equation (7) based on minimal variance theory.

(6) $$\{\begin{array}{l} X_{\sigma 1}=\left[\begin{array}{cc} 1 & -10\log_{10}d_{1} \end{array}\right]\left[\begin{array}{c} A\\ n \end{array}\right]-R_{d1}\\ \cdots \\ X_{\sigma m}=\left[\begin{array}{cc} 1 & -10\log_{10}d_{m} \end{array}\right]\left[\begin{array}{c} A\\ n \end{array}\right]-R_{dm} \end{array}$$

(7) $$\left[\begin{array}{c} \hat{A}\\ \hat{n} \end{array}\right]={\left({\left[\begin{array}{c} \begin{array}{cc} 1 & -10\log_{10}d_{1} \end{array}\\ \cdots \\ \begin{array}{cc} 1 & -10\log_{10}d_{m} \end{array} \end{array}\right]}^{T}P\left[\begin{array}{c} \begin{array}{cc} 1 & -10\log_{10}d_{1} \end{array}\\ \cdots \\ \begin{array}{cc} 1 & -10\log_{10}d_{m} \end{array} \end{array}\right]\right)}^{-1}{\left[\begin{array}{c} \begin{array}{cc} 1 & -10\log_{10}d_{1} \end{array}\\ \cdots \\ \begin{array}{cc} 1 & -10\log_{10}d_{m} \end{array} \end{array}\right]}^{T}P\left[\begin{array}{c} R_{d1}\\ \cdots \\ R_{dm} \end{array}\right]$$

### 4.3. Radio Map Construction based on the Built Model

With the help of the estimated optimal parameters $\hat{A}$ and $\hat{n}$, a log-distance path loss model of an AP in a small area can be obtained. In previous studies, if the coordinates of IP are known, the distance between the IP and AP and the RSS value can be calculated by Equation (3). They ignore the characteristics of RSS loss, RSS fluctuation, and RSS threshold, which are common in a large-scale building. The detailed algorithm is described in Algorithm 1.

**Algorithm 1.** Radio map construction based on adaptive path loss model interpolation1. **Input:** sparse RPs in the whole testbed area, APs’ locations, IPs, *m*, *σ*2. **Output:** interpolated radio map3. N_RP_ = Num(RPs), N_AP_ = Num(APs), N_IP_ = Num(IPs);4. **for**
*i* = 1 to *N_IP_*
**do**5.  P_IP_ = IPs(*i*), loc_IP_ = P_IP_.loc;6.  **for**
*j* = 1 to *N_RP_*
**do**7.   Poly = Polygon(RPs(*j*), RPs(*j* + 1), …, RPs(*j* + *m* − 1));8.   **if** P_IP_ IsIn(Poly) **then**9.    MAC_Temp = Unique_MAC(RPs(*j*), RPs(*j* + 1), …, RPs(*j* + *m* − 1));10.    traverse these *m* RPs, if one RP does not have MAC in MAC_Temp, the pair of MAC and −100 are stored in the RP;11.    **for** k = 1 to *Num*(MAC_Temp) **do**12.     MAC = MAC_Temp(k), loc_AP_ = AP.loc;13.     [A, n] = LeastSquares(loc_AP_, RPs(*j*), RPs(*j* + 1), …, RPs(*j* + *m* − 1));14.     RSS = Model(loc_IP_, loc_AP_, *A*, *n*, *σ*);15.     **if** RSS < −100 **then**16.      RSS = −100;17.     **elseif** RSS > −30 **then**18.      RSS = −30 + Randn(*σ*);19.     **end if**20.      P_IP_.add(MAC, RSS);21.    **end for**22.   **end if**23.   **if**
*j* + *m* >= *N_RP_*
**then**24.    break;25.   **end if**26.  **end for**27. **end for**28. Combine(RPs, IPs)

In general, RSS fingerprints at RPs are characterized by several tens of static samples. This process can accurately describe the RSS distribution at RPs and detect APs as many as possible. If some APs are undetectable at an RP, the corresponding RSS can be set as –100 dBm. To reduce the computation, we only replace the missing RSS value with –100 dBm step by step in the small area rather than the whole testbed area. The big advantage is that only several path loss models are built instead of models of all APs in the whole testbed area.

Because of the dynamic and complicated wireless environment, the RSS fluctuation appears at a static location all the time. Thus, the RSS noise should be introduced in RSS estimation by path loss model.

To the best of our knowledge, RSS value detected by smartphone is usually less than −30 dBm and larger than −100 dBm. Therefore, the threshold restraint should be added.

In the Algorithm, Polygon() denotes that a polygon is formed by *m* locations of RPs, then the polygon can be used for adjusting whether IP is in it by IsIn(). If the IP is in the polygon, we can utilize these *m* RPs to build path loss model based on least squares. By using Unique_MAC(), we can get all unique MAC of detectable APs in a small area. The MAC_Temp is used for building path loss models of all hearable APs. If one of these *m* RPs does not include a MAC in MAC_Temp, the pair of MAC and -100 will be added in the RP. LeastSquares() utilizes the location of an AP, locations and RSS values of the AP at *m* RPs to estimate *A* and *n*. Then, Model() can calculate the RSS value of the AP at IP by (3) with a random noise by Randn(). IP adds the pair of MAC and RSS after a little adjustment. If the calculated RSS value is larger than −30 dBm or less than −100 dBm, the corresponding adjustment are provided from the 15th line to 19th line of the Algorithm. Randn() can get a random noise with the standard deviation *σ*. After traversing all IPs, interpolated radio map can be obtained through combining RPs and IPs by Combine().

## 5. Experiments and Discussion

This section shows the experimental testbed and evaluates the RSS estimation and positioning performance of radio maps interpolated by IDW, Kriging and our proposed methods, as well as the complete manual radio map.

To evaluate the positioning performance of different radio maps fairly, the weighted K-nearest neighbor (WKNN) method is introduced without clustering. In our experiments, K is set as 5. We adopt mean error (ME), root mean square error (RMSE) as a quantitative index for representing positioning accuracy.

### 5.1. Experimental Testbed Setup

**Figure 2:**
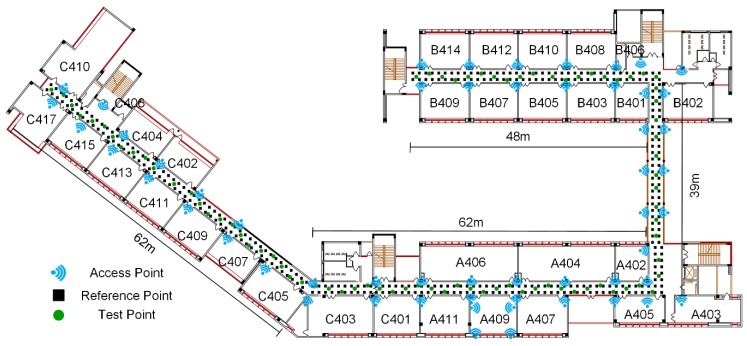
The layout of the experimental testbed, where the blue signal symbol denotes Wi-Fi access point, the black solid rectangle refers to the reference point, and the green solid point is test point.

Figure 2 is the layout of the experimental testbed, occupying the whole 4th floor of School of Environment Science and Spatial Informatics, China University of Mining and Technology (CUMT), Xuzhou, China. The area of the testbed is almost 3200 m^2^. The main experimental scene is the corridor, whose total length is about 211 m and width is larger than 2.4 m. It’s easy to count out how many APs are displayed with the blue signal symbol in this picture. There are 56 TP-Link 2.4 GHz APs pre-installed with the height of 3m. Among them, only four APs are deployed in room A409. During RSS collections, pedestrians appear with normal activities. Most of APs are installed in corridors, where disturbance caused by pedestrians are relatively little. In order to avoid the pedestrian impact on RSS, experiments are only conducted in corridors.

352 RPs are displayed in Figure 2 with a black solid rectangle. The distance between two adjacent RPs is about 1.2 m. At each RP, 60 samples are conducted with the frequency of 1 Hz along with the direction of the building. Eight postgraduates collect RSS fingerprints at these 352 RPs with eight smartphones in different brands or types, such as Mi. 6, Mi. 5X, Huawei Mate 8, and Samsung Galaxy S7. RSS samples are collected at one RP by using a smartphone, while at another RP RSS samples are collected by the other one. These 352 RPs with RSS fingerprints from eight smartphones are merged and then trained for constructing the complete manual radio map with the interval of 1.2 m for the following comparisons. It is worthy to note that the simple RSS data merge are at the risk of RSS differences caused by device heterogeneous. Lots of research is proposed to deal with this issue, such as SSD [30,31], DIFF [32], and methods detailed in our previous paper [24]. Because the device heterogeneous is one of the biggest challenges for indoor positioning and indoor navigation, and the mentioned research behave well, we ignore this effect in our experiments to focus on the method of radio map construction.82 test points (TPs), green solid points, are designed to evaluate the performance of different radio map. The number of TPs is about 23% of RPs. They are evenly distributed in the experimental testbed area. At each TP, 10 samples are conducted with the same frequency and direction as at RPs. Therefore, there are 820 samples for evaluating positioning accuracy of the radio map in total.

In order to study how to construct radio map efficiently and accurately, sparse RPs at different intervals ranging from 2.4 m to 18 m are selected from the complete manual radio map. The interval gradually increases by 1.2 m. The selected sparse RPs are used for building radio maps by different interpolation methods. Due to the variety of indoor structure and layout, RPs should be selected for radio map construction at the start and end of both structures and layouts. In addition, the length of certain a structure may be not the euploid number of the interval, the RPs, who are regarded as the start of this partition less than an interval, should be also selected. Thus, the number of selected sparse RPs is not decrement by a fixed number. The number of sparse RPs with different sampling intervals are shown in Table 2.

**Figure 3:**
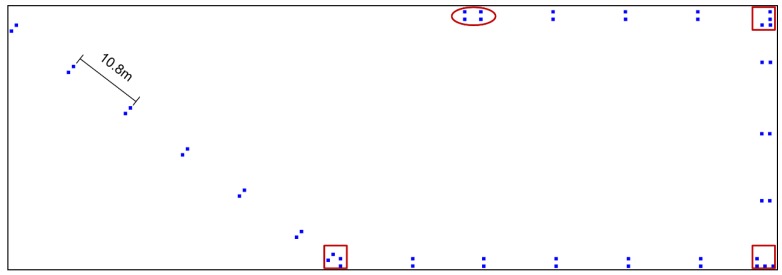
The distribution of selected sparse RPs with the interval of 10.8 m, where the red square rectangle denotes the start and end of adjacent structures, the red ellipse refers to the partition less than the interval.

Taking the interval of 10.8 m as an example, Figure 3 is the distribution of selected RPs, there are 50 RPs displayed by a blue solid rectangle. The majority of intervals are 10.8 m. The experimental testbed includes four corridors, the three red square rectangle denote the start and end of adjacent structures. The corridor at the top of this layout is about 48 m, the fifth range is less than 10.8 m, and this part in red ellipse should be added as sparse RPs.

### 5.2. Experiments and Results

In this subsection, interpolated radio maps are constructed based on these 14 groups sparse RPs through IDW, Kriging and our proposed path loss model interpolation methods, respectively. Comparisons are conducted between these interpolated radio maps and the complete manual one with respect to RSS estimation and localization performance. For convenience in expression, the obtained three types of interpolated radio maps are simplified as complete manual radio map (Manual RM), IDW interpolated radio map (IDW Interp_RM), Kriging interpolated radio map (Kriging Interp_RM) and path loss model interpolated radio map (PL Interp_RM).

#### 5.2.1. The Effect of RSS Noise on the Constructed Radio Map

In our proposed radio map construction method, we have introduced RSS noise in Equation (3) to estimate RSS received at one RP, where the wireless signal may suffer from multiple path effect, obstacles sheltering, and pedestrians. In our experiment, we regard RSS noise as Gaussian white noise, and adopt a random function to generate random noise with the standard deviations (*σ*), which usually ranges from 1 to 7. Based on different intervals and RSS noise, 98 PL Interp_RM will be constructed by our proposed method. To characterize the similarity between Manual RM and these PL Interp_RM, the correlation coefficients are calculated by Pearson correlation coefficient formula, as is shown is Equation (8), where ρ(X,Y) denotes correlation coefficient, X and Y are independent variables, *E*() denotes the expectation, *μ* refers to mean value, *σ* is standard deviation.

(8) $$\rho \left(X,Y\right)=\frac{E\left[\left(X-\mu_{X}\right)\left(Y-\mu_{Y}\right)\right]}{\sigma_{X}\sigma_{Y}}$$

**Figure 4:**
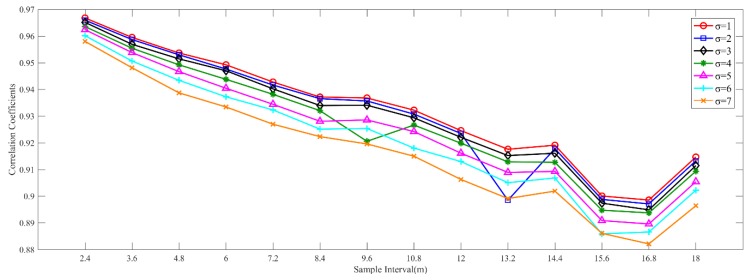
The correlation coefficients between the complete manual radio map and adaptive path loss model interpolated radio maps, which are generated by sparse RPs with different intervals and standard deviations.

**Figure 5:**
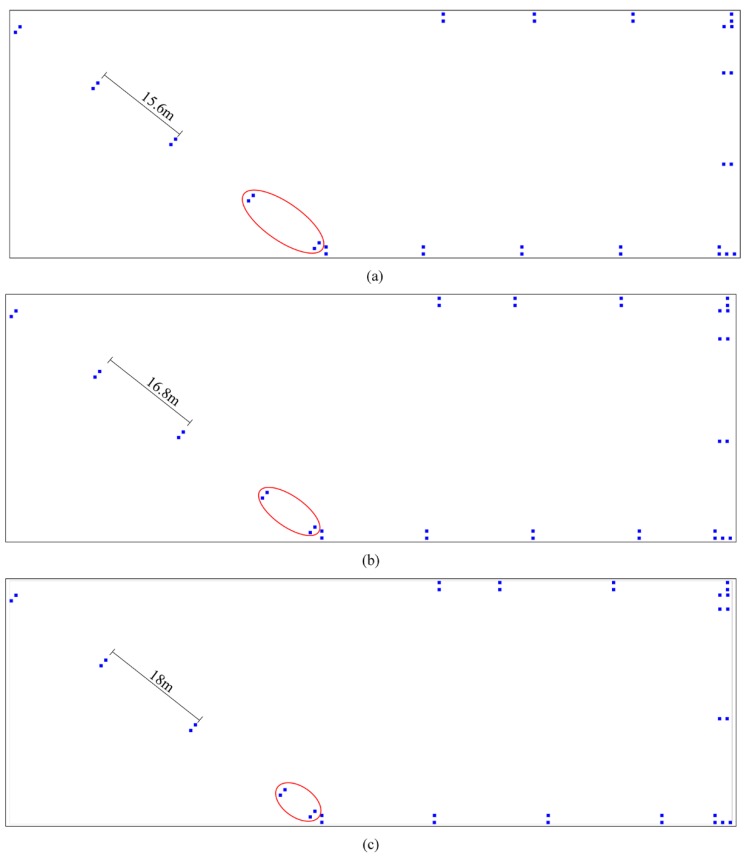
The distribution of selected sparse RPs with the interval of 15.6 m, 16.8 m and 18 m, respectively, where the red ellipse refers to the partition less than the interval, (**a**) the distribution of selected RPs with the interval of 15.6 m, (**b**) the distribution of selected RPs with the interval of 16.8 m, (**c**) the distribution of selected RPs with the interval of 18 m.

**Figure 6:**
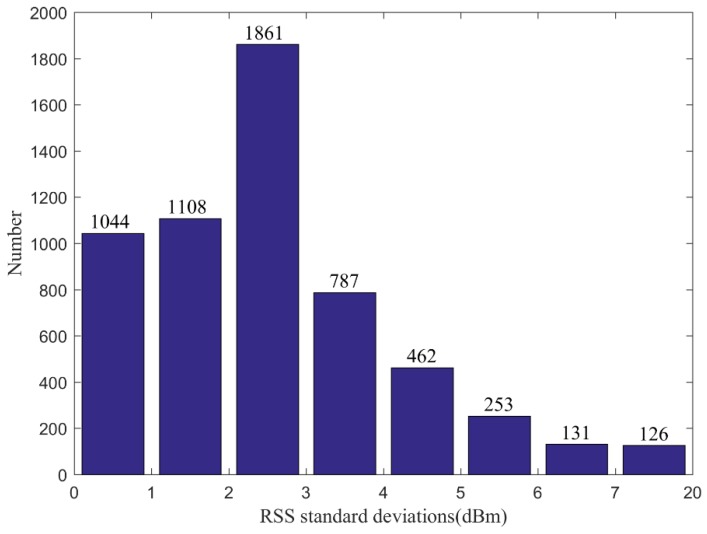
The statistics of RSS standard deviation values at 352 RPs.

Figure 4 shows the correlation coefficients between Manual RM and these interpolated radio maps, which are generated by sparse RPs with different intervals and RSS noise. From this picture, we can find that the correlation coefficient decreases gradually with the increase of interval, as well as the standard deviation. It seems to be abnormal when the interval is set as 18 m. The correlation coefficients are larger than those with the interval of 15.6 m and 16.8 m when the standard deviation is a fixed value. In our opinion, the reason for this is that the distance of the part not enough 18 m at the end of a building structure is smaller than those not enough both 15.6 m and 16.8 m. As is shown in Figure 5a–c, the distributions of selected RPs with the intervals of 15.6 m, 16.8 m and 18 m, respectively. The selected part in the red ellipse in (c) is smaller than those in (a) and (b), as well as unselected parts at the end of other corridors. The interpolated RSS values in these parts in (c) are likely to be more accurate than (a) and (b), which cause larger correlation coefficients when the interval is 18 m. When the standard deviation is set as 1, all the correlation coefficients between two types of radio maps are biggest, no matter which interval is selected for constructing the interpolated radio map. When the standard deviation is 7, all the correlation coefficients are almost minimal. When the standard deviations are not larger than 3, all the correlation coefficients are larger than 0.9. By using the statistics of RSS standard deviations at 352 RPs, we find that the number of standard deviations between 2 and 3 is largest, 1861, shown in Figure 6. Although choosing the standard deviation as 1 will get good performance of RSS estimations and positioning, we set the standard deviation as 3 in our adaptive path loss model interpolated radio map construction for practical reasons.

#### 5.2.2. The RSS Differences between Manual Radio Map and Interpolated Ones.

To calculate the RSS differences, all missing RSS values are set as −100 dBm in all interpolated radio maps and the complete manual one. Then, RSS differences can be obtained by the RSS fingerprints in manual radio map minus corresponding RSS values in each interpolated radio map. The statistics of calculated absolute RSS differences between manual radio map and interpolated ones are shown in Table 3, Table 4 and Table 5 respectively. From these three tables, we can find that fluctuations become higher as the interval increases. When the interval is small, the probabilities in the same absolute RSS differences ranges are very close. However, when the interval enlarges larger than 10.8 m, the probabilities concerning the IDW interpolated radio map is smaller than the other radio maps. The IDW interpolated radio maps behave poor in RSS interpolation, while the Kriging interpolated radio maps behave best.

**Figure 7:**
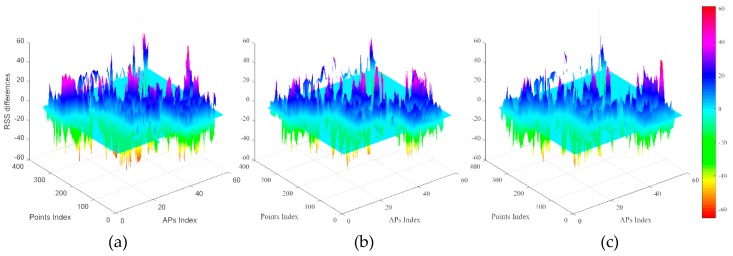
RSS differences between manual radio map and (**a**) IDW Interp_RM, (**b**) Kriging Interp_RM, (**c**) PL Interp_RM constructed based on the sparse RPs with the interval of 10.8 m.

Figure 7 shows RSS differences between the complete manual radio map and IDW Interp_RM, Kriging Interp_RM, PL Interp_RM constructed based on the sparse RPs with the interval of 10.8m. RSS differences are calculated by the complete manual radio map minus the interpolated radio map with the same MAC. From these pictures, the performance of different interpolated radio maps can be found on positive or negative RSS differences. Different colors denote different RSS differences. Red indicates big absolute RSS differences. The maximum height of bulges in Figure 7c is lower than in both Figure 7a,b under the zero differences plane, because the number of red bulges in Figure 7c is least. This illustrates that the interpolated radio map based on our proposed method has a good performance in strong RSS value interpolation. The maximum height of bulges in Figure 7b is lower than in both Figure 7a,c, over the zero RSS differences plane. In addition, there are many large RSS differences, such as 60dBm. These large RSS differences may be caused by replacing missing RSS value with a constant value (−100 dBm).

It is worthy to note that the RSS fingerprints at two nearby RPs are usually similar in Kriging interpolated radio maps. It is the reason for why Kriging interpolated radio maps behave higher correlation coefficients and higher probabilities with small RSS differences intervals.

#### 5.2.3. The Comparisons of Positioning Accuracy

**Figure 8:**
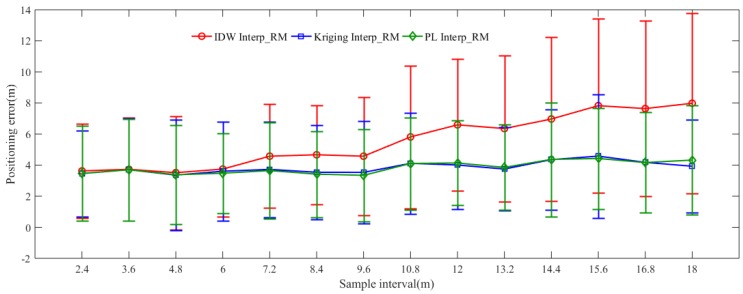
Localization accuracy of three interpolated radio maps generated by sparse RPs with different intervals.

Figure 8 shows the ME and RMSE of three interpolated radio maps generated by sparse RPs with different intervals. The red, blue and green curves represent positioning accuracy of IDW Interp_RM, Kriging Interp_RM and PL Interp_RM, respectively. The middle point denotes ME, while the range in vertical direction means RMSE around ME. Taking the interval of 10.8 m as an example, the position of the red circle is higher than of blue rectangle and green diamond, the positions of blue rectangle and green diamond are very close; and the range in red is larger than in blue and green, the green range is the smallest. In other words, the IDW Interp_RM, constructed based on the sparse RPs with the interval of 10.8 m, gets the worst positioning accuracy, while the PL Interp_RM gets the best. It can be seen from the figure that the positioning accuracy of PL Interp_RM outperforms IDW Interp_RM and Kriging Interp_RM at the same interval overall.

Table 6 shows comparisons of positioning accuracy among the complete manual radio map and IDW Interp_RM, Kriging Interp_RM, PL Interp_RM with different intervals. We can see that the ME of the complete manual radio map with the interval of 1.2 m is 3.341 m, the RMSE is 3.026 m. When the intervals range from 2.4 m to 3.6 m, the positioning errors are basically identical for the complete manual radio map, IDW Interp_RM, Kriging Interp_RM and PL Interp_RM. In other words, if the sample interval is smaller than 3.6 m, it is no need to interpolate RSS fingerprints for dense radio map. Kriging Interp_RM and PL Interp_RM can obtain the same or better positioning accuracy when the interval is ranging from 2.4 m to 9.6 m as the complete manual radio map with the interval of 1.2 m. Similarly, taking the interval of 10.8 m as an example, the ME of IDW Interp_RM is about 5.8 m, and the RMSE is around 4.6 m; the ME of Kriging Interp_RM is about 4.1 m, and the RMSE is around 3.2 m; while the ME of PL Interp_RM is about 4.1 m, and the RMSE is around 3 m. The performance of Kriging Interp_RM and PL Interp_RM are similar, while the performance of IDW Interp_RM is the worst, even worse than the complete manual radio map with the same interval. It is obvious that the PL Interp_RM gets a better positioning accuracy than IDW Interp_RM and Kriging Interp_RM when the interval is less than 12 m. The Kriging Interp_RM gets the best positioning accuracy when the interval is larger than 12 m. However, the computational complexity of Kriging interpolation method is O(n^3) [33], while that of the proposed method is O(n^2) [34], where n refers to the size of the observed dataset. Thus, the PL Interp_RM behaves well in the performance of positioning accuracy in general.

We can also find a rough relationship between positioning accuracy from Table 6 and the number of sparse RPs from Table 2. In general, the larger the interval between adjacent RPs is, the worse the positioning accuracy are achieved. However, Kriging Interp_RM and PL Interp_RM get the same or better positioning accuracy as the complete manual radio map when the interval is no larger than 9.6 m. But above all, the radio map with the same positioning accuracy is interpolated based on sparse RPs at the maximum interval of 9.6 m, and the number of RPs in this sparse RPs is only 54, while the number of RPs in the complete manual radio map is 352.

**Figure 9:**
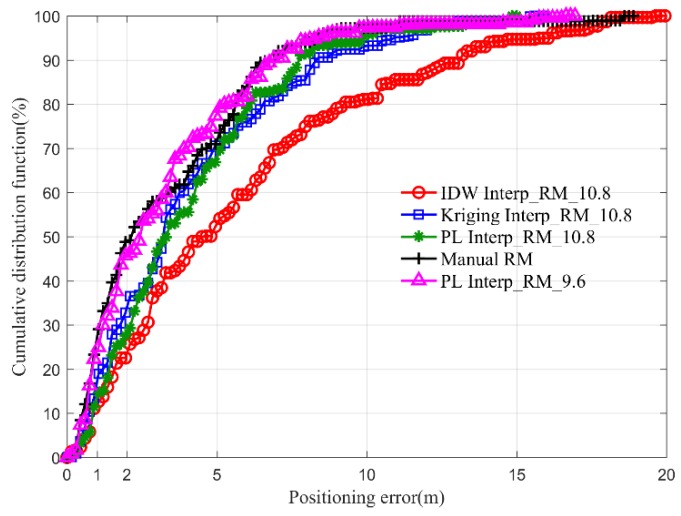
Cumulative distribution of localization errors of different interpolated radio maps generated by sparse RPs with different intervals and manual radio map.

Figure 9 shows the cumulative distribution of localization error of three interpolated radio maps with different intervals and the complete manual radio map. There are five curves displaying in this figure with respect to 4 types of radio maps. The black curve with plus denotes the cumulative positioning error of the complete manual radio map. The pink curve with triangle denotes the cumulative positioning error of PL Interp_RM, which is built based on the sparse RPs with the interval of 9.6 m. The red circle, blue rectangle and green star curves denote the cumulative positioning error of IDW Interp_RM, Kriging Interp_RM and PL Interp_RM, which are constructed based on the sparse RPs with the interval of 10.8 m, respectively. From this picture, we can see that the accuracy of the complete manual radio map is better than of IDW Interp_RM, Kriging Interp_RM and PL Interp_RM with the interval of 10.8 m. But those of the complete manual radio map is worse than PL Interp_RM with the interval of 9.6 m. As in the previous mentioned, the PL Interp_RM has a better performance of strong RSS estimation. The strong RSS usually plays a more important role than weak one in fingerprinting localization. Therefore, it is the reason why PL Interp_RM has a better localization performance than the complete manual radio map.

## 6. Discussion

We explored the fast and accurate radio map construction with many APs in a large-scale building, about 3200 m^2^ on a single floor, considering RSS fluctuation, RSS loss and RSS threshold. The suitable sample interval between two nearby RPs is 9.6 m, which can minimize the construction workload with the same positioning accuracy as the complete manual radio map with the interval of 1.2 m. The number of sparse RPs with the interval of 9.6 m is only 54, while the complete one has 352. The proposed method can reduce almost 85% workload and time to construct a radio map with a moderate positioning accuracy which can meet the common localization requirements.

The Kriging Interp_RM behaves best in terms of RSS differences and correlation coefficients. Our proposed PL Interp_RM is poorer than the Kriging Interp_RM in terms of RSS estimation of the whole area. The proposed PL Interp_RM gets the best RSS estimation in terms of strong RSS. This is the reason why the Kriging Interp_RM with a better RSS estimation behaves poorer positioning performance than the proposed PL Interp_RM with a poor RSS estimation in the whole area.

The proposed method can also be used for a radio map update. If the radio map should be updated, a new interpolated radio map could be re-constructed rapidly after RSS fingerprints are collected at some RPs. Although our proposed path loss model interpolated radio map can achieve better performance of localization accuracy relative to IDW and Kriging interpolated methods at the same interval. It has still some disadvantages. Especially, the method is conducted with the knowledge of APs’ locations. The adopted log-distance path loss model may be not able to reflect the distance between AP and mobile device, because of ignoring walls, floors, and human beings’ attenuation, as well as multipath interference, diffractions and other effects. In the future, we will adopt the more precise path loss model to generate RSS fingerprint, such as the multi-wall model taking wall attenuation factor (WAF) and floor attenuation factor (FAF) into account. Moreover, we will try to explore the method without the knowledge of APs’ locations by utilizing some other parameters estimation methods to construct radio map, such as Gaussian process regression, partial least square squares. Furthermore, we will validate the effectiveness of the improved method in different environments, e.g., shopping malls, halls, and theatres.

## 7. Conclusions

This study proposes a method of fast radio map construction by using static crowdsourcing RSS fingerprints and adaptive path loss model interpolation in a large-scale building, considering RSS fluctuation, RSS loss and RSS threshold. By comparing with IDW and Kriging interpolation methods, we find that the interpolated radio map based on our proposed method outperforms other interpolated methods in positioning performance, as well as the complete manual radio map at the interval of 1.2 m. 85% workload and time can be saved to construct a radio map with a satisfactory positioning accuracy by our proposed method.

## Figures and Tables

**Table 1:** Comparisons of different methods for radio map construction.

Methods	System Name	Algorithm and Requirements	Accuracy of RM	Testbed Area
crowdsourcing	Redpin [5]	label position by user, indoor map	room level (90%)	26 rooms
	Molé [6]	Kernel, accelerometer	room level (91%)	3-floor building
	FreeLoc [7]	relative RSS comparison	<3 m	a laboratory, a corridor
SLAM	WiFi-SLAM [8]	GP-LVM, initial Isomap model	3.97 m (ME)	250–500 m(traces)
	SignalSLAM [9]	least square, PDR, GraphSLAM, landmarks, accelerometer, gyroscope, magnetometer	<16.5 m (MD)	200 m × 160 m
inertial sensors	Zee [10]	DR, augmented particle filter, indoor map, accelerometer, gyroscope, magnetometer	1.2 m (50%), 1.8 m (80%)	65 m × 35 m
	LiFS [11]	DR, feature extraction, indoor map, accelerometer	5.88 m (ME)	70 m × 23 m
	WILL [12]	PDR, K-means, accelerometer	room level (86%)	70 m × 23 m
semi-supervised learning	[13]	manifold learning, path loss model, partial RPs, APs’ locations, indoor map	3.8 m (ME); 2.4 m (ME)	40 m × 30 m, 5 APs; 40 m × 20 m, 4 APs
	[14]	Co-Forest, partial RPs	3.65 m (ME)	800 m^2^, 30 APs
unsupervised learning	WRM [15,16]	HMM, EM, memetic algorithm, path loss model, indoor map, APs’ locations	around 3 m (ME)	80 m × 32 m, 30 APs
path loss model	ARIADNE [17]	ray tracing, path loss model, simulated annealing algorithm, APs’ location, partial RPs, indoor map	3 m (ME), 2.5 m (STD)	65 m × 48 m, 5 APs
	[18]	multi-wall path loss model, APs’ location, indoor map, parameters setting	1.2 m (ME)	480 m^2^, 3 APs
interpolation	[19]	IDW	5 ~ 20m(ME)	150 m × 60 m, 316 2.4 GHz APs, 106 5 GHz APs
	[20]	Kriging	1.12 m (ME)	9.5 m × 2.5 m, 9 APs
	[21]	cubic spline	2.82 m (Best)	5 rooms, 4 APs

**Table 2:** The number of sparse RPs with different sampling intervals.

Intervals (m)	1.2	2.4	3.6	4.8	6	7.2	8.4	9.6	10.8	12	13.2	14.4	15.6	16.8	18
Number	352	180	126	96	80	70	62	54	50	46	42	42	36	36	36

**Table 3:** The statistics probabilities of absolute RSS differences ranges between manual radio map and the IDW interpolated ones.

Intervals	2.4	3.6	4.8	6	7.2	8.4	9.6	10.8	12	13.2	14.4	15.6	16.8	18
<3 dBm	0.888	0.851	0.822	0.804	0.803	0.786	0.775	0.76	0.752	0.748	0.754	0.735	0.739	0.724
<5 dBm	0.918	0.888	0.868	0.851	0.848	0.831	0.822	0.809	0.796	0.793	0.796	0.778	0.778	0.764
<10 dBm	0.962	0.948	0.937	0.928	0.919	0.91	0.9	0.889	0.88	0.875	0.873	0.861	0.86	0.848

**Table 4:** The statistics probabilities of absolute RSS differences ranges between manual radio map and the Kriging interpolated ones.

Intervals	2.4	3.6	4.8	6	7.2	8.4	9.6	10.8	12	13.2	14.4	15.6	16.8	18
<3 dBm	0.89	0.856	0.828	0.808	0.813	0.799	0.787	0.778	0.772	0.773	0.777	0.757	0.763	0.749
<5 dBm	0.921	0.895	0.876	0.863	0.86	0.852	0.844	0.833	0.831	0.828	0.826	0.807	0.816	0.803
<10 dBm	0.964	0.954	0.945	0.94	0.933	0.93	0.926	0.916	0.917	0.912	0.909	0.897	0.904	0.897

**Table 5:** The statistics probabilities of absolute RSS differences ranges between manual radio map and the adaptive path loss model interpolated ones.

Intervals	2.4	3.6	4.8	6	7.2	8.4	9.6	10.8	12	13.2	14.4	15.6	16.8	18
<3 dBm	0.887	0.849	0.825	0.808	0.808	0.79	0.78	0.774	0.766	0.765	0.765	0.748	0.754	0.747
<5 dBm	0.912	0.883	0.865	0.851	0.849	0.835	0.825	0.82	0.812	0.813	0.812	0.793	0.797	0.792
<10 dBm	0.955	0.942	0.934	0.927	0.923	0.915	0.91	0.903	0.899	0.894	0.893	0.878	0.882	0.881

**Table 6:** Comparisons of localization errors among different radio maps (m).

Intervals	Manual RM		IDW Interp_RM		Kriging Interp_RM		PL Interp_RM	
	ME	RMSE	ME	RMSE	ME	RMSE	ME	RMSE
1.2	3.341	3.026	--	--	--	--	--	--
2.4	3.54	3.083	3.627	3.015	3.459	2.778	3.468	3.043
3.6	3.883	3.264	3.731	3.317	3.702	3.293	3.7	3.273
4.8	4.078	3.466	3.515	3.649	3.358	3.572	3.382	3.181
6	3.977	3.223	3.746	3.045	3.609	3.176	3.48	2.57
7.2	4.709	4.646	4.576	3.343	3.726	3.069	3.65	3.091
8.4	4.956	5.128	4.667	3.181	3.542	3.016	3.42	2.764
9.6	4.61	4.193	4.576	3.813	3.533	3.283	3.344	2.952
10.8	5.57	5.238	5.812	4.587	4.116	3.242	4.106	2.97
12	4.86	3.625	6.594	4.238	4.008	2.861	4.137	2.721
13.2	5.245	3.854	6.354	4.684	3.745	2.683	3.853	2.746
14.4	6.012	4.446	6.969	5.261	4.353	3.246	4.371	3.672
15.6	6.3	5.429	7.827	5.603	4.582	3.985	4.428	3.25
16.8	5.8	4.998	7.64	5.66	4.178	3.247	4.165	3.233
18	5.885	4.991	7.982	5.787	3.929	3.002	4.326	3.524
